# Dynamic transcriptome profiling towards understanding the morphogenesis and development of diverse feather in domestic duck

**DOI:** 10.1186/s12864-018-4778-7

**Published:** 2018-05-24

**Authors:** Jing Yang, Yanhua Qu, Yuan Huang, Fumin Lei

**Affiliations:** 1 Key Laboratory of the Zoological Systematics and Evolution, Institute of Zoology, the Chinese Academy of Sciences, Beijing, 100101 China; 2 School of Environmental Science and Engineering, Shaanxi University of Science and Technology, Xi’an, 710021 China; 3 Co-Innovation Center for Qinba Regions’ Sustainable Development, School of Life Sciences, Shaanxi Normal University, Xi’an, 710062 China; 4 Center for Excellence in Animal Evolution and Genetics, Chinese Academy of Sciences, Kunming, 650223 China

**Keywords:** Plumulaceous feather, Flight feather, Differentiation, Transcriptome, Duck

## Abstract

**Background:**

Feathers with complex and fine structure are hallmark avian integument appendages, which have contributed significantly to the survival and breeding for birds. Here, we aimed to explore the differentiation, morphogenesis and development of diverse feathers in the domestic duck.

**Results:**

Transcriptome profiles of skin owing feather follicle from two body parts at three physiological stages were constructed to understand the molecular network and excavate the candidate genes associated with the development of plumulaceous and flight feather structures. The venn analysis of differentially expressed genes (DEGs) between abdomen and wing skin tissues at three developmental stages showed that 38 genes owing identical differentially expression pattern. Together, our data suggest that feather morphological and structural diversity can be possibly related to the homeobox proteins. The key series-clusters, many candidate biological processes and genes were identified for the morphogenesis, growth and development of two feather types. Through comparing the results of developmental transcriptomes from plumulaceous and flight feather, we found that DEGs belonging to the family of WNT, FGF and BMP have certain differences; even the consistent DEGs of skin and feather follicle transcriptomes from abdomen and wing have the different expression patterns.

**Conclusions:**

Overall, this study detected many functional genes and showed differences in the molecular mechanisms of diverse feather developments. The findings in WNT, FGF and BMP, which were consistent with biological experiments, showed more possible complex modulations. A correlative role of HOX genes was also suggested but future biological verification experiments are required. This work provided valuable information for subsequent research on the morphogenesis of feathers.

**Electronic supplementary material:**

The online version of this article (10.1186/s12864-018-4778-7) contains supplementary material, which is available to authorized users.

## Background

Avian feathers are branched integumentary appendages that play important roles in flight, protection, heat retention, communication, and mate attraction, due to they could form different structures in various body parts or at different phases during their life stages [1]. The prosperous feather diversities have contributed significantly to the rapid and extensive radiation of birds to become the dominant vertebrate [2]. As the most intricate integumentary appendages with diversiform shapes, arrangements and pigmentations, feathers are regarded as an excellent model for evolutionary and developmental biology research [3].

Feathers are divided into three main types of contour feathers (pennaceous), downy feathers (plumulacuous) and filoplumes. Contour feathers not only include the ordinary body contour feathers, but also flight feathers (remiges) and rectrices. Feather branching at three levels, the rachis, barbs and barbules offer more opportunities for diversity. Modulating their size, angle and symmetry generates complex feather forms. The downy feathers are radial symmetry, and their barbules do not possess the hooklets, hence a fluffy structure could be formed to keep body warm [4]. After processing, the downy feather is well suited for the production of down jacket. While strong flight feathers are left-right asymmetry, which have a larger pennacuous vane (barbules interlock with each other through hooklets), and a longer and thicker rachis for insertion deeper into the follicle. The structure of the flight feathers could anchor more securely to hold its aerodynamic function [5].

The morphogenesis of feather is initiated by the interplay of epithelia and subjacent mesenchayme and usually involves a series of dynamic cellular processes [2]. There are several key molecules that controlled the fundamental aspects of these processes, including growth factors and their receptors, cell adhesion molecules and their ligands, signal transduction molecules and transcription factors [6–10]. Recent studies of feather morphogenesis have concentrated on the molecular mechanisms underlying placode induction and feather bud formation. Little attention has been paid to the regulatory networks that pattern and define the morphogenesis of the elaborate feather structure. Only a handful of signal transduction molecules, cell junctions, feather keratins and other few genes have been reported to possibly involved in the formation of diverse feathers. Previous studies revealed that BMP and SHH signaling are involved in regulating the formation and balance among the rachis and barbs [7]. A feather morphogenesis model suggests that plumulaceous feather structure evolved by the establishment of activator-inhibitor interactions between SHH and BMP2 signaling in the basal epithelium of the feather germ [11]. In addition, the mis-expressed of *BMP4* would enhance the rachis formation and barbs fuse. When noggin (a BMP antagonist) is mis-expressed, the rachis is split and increased barb branching ensues [7]. Perturbing the gradient of *WNT3A* converts bilaterally symmetric feathers into radially symmetrical feathers [12]. The complex branching pattern of feathers may derive from the establishment of specific cell junctions among barb/barbules cells. Gap junctions serve in cell communication while tight junctions stabilize the complex branching of keratinized feather cells [13]. Feathers consist mainly of flexible corneous materials made of α- and β-keratin multigene families [14]. Ng et al. [15] suggested that feather keratins on chromosome 2 of *Gallus gallus* may have significant effects on the formation of stiff feather structures, and feather keratin on chromosome 25 may be required for softer textures. Furthermore, the crest phenotype is caused by a cis-acting regulatory mutation underlying the ectopic expression of *HOXC8* [16].

Domestic duck feathers provide an excellent system for studying the formation and development of morphological complexity because it has diverse forms in different body parts or at different phases during its whole life. Comprehensive cataloguing of gene expression changes of skin and follicle tissues of domestic duck at different physiological stages or body parts would be beneficial to further understand the complex structure of feathers. The availability of transcriptomic analysis provides an excellent opportunity to study gene expression patterns that potentially account for the diverse feather forms [17].

Our objective was to analyze the biological information regarding the transcriptional profiles of skin and follicles to further identify differentially expressed genes and key gene regulatory networks affecting the differentiation and development of diverse feathers through the domestic duck model. We characterized and quantified mRNAs that are expressed in the skin and follicles during feather development in Cherry Valley ducks at two parts of body (abdomen and inner side of the wing) during three stages of feather development. Two body parts of skin and follicles were selected to represent different feather types. We made three analyses: 1) the developmental transcriptomes of abdomen skin and follicles for capturing the functional genes and molecular events accounting for the morphogenesis and development of plumulaceous feather; 2) the developmental transcriptomes of inner side of the wing skin and follicles for obtaining the functional genes and molecular events associated with the morphogenesis and development of flight feather. 3) the comparisons of abdomen and wing skin transcriptomes at the same physiological stages for understanding the differentiation of plumulaceous feathers and flight feathers; Our work has firstly presented a large-scale genomics resource for understanding the evolution and morphogenesis of various feather types.

## Results

### Overview of RNA-Seq data

A total of 12 libraries were sequenced from skin owing feather follicle tissues of six groups (*n* = 2 for each), including skin owing feather follicle tissues of the abdomen and inner side of the wing at three different physiological stages (3-day-old, 27-day-old and 6-month-old), representing the early growth plumulaceous feathers (EP), middle growth plumulaceous feathers (MP), late growth plumulaceous feathers (LP), early growth flight feathers (EF), middle growth flight feathers (MF) and late growth flight feathers (LF), respectively. About 4.00–4.36 Gb raw reads were produced for each library. After discarding low-quality reads, RNA-seq yielded from 42,914,954 to 47,459,658 clean reads with average about 97% Q20 bases for each sample, which were used for all further expression analysis. Among the total number of clean reads from 12 samples, 69.45 to 80.22% were successfully mapped against the reference Peking duck genome (Table 1). The percentage of the unique mapping reads is approximate 71.93% in each sample. Two issues that may be considered as possible reasons for the lower mapped rates are the draft assembling and annotation of Peking duck genome and evolutionary divergence between Cherry Valley duck and Peking duck. Gene structure analysis was performed for each group (Additional file 1: Figure S1), most of the clean reads (43.63–54.50%) were aligned to the exon regions of the reference genome, followed by the number of the clean reads matched to the 3’UTR and intergenic regions. Correlation of transcript expression level is a crucial indicator for the reliability of the experimental results and the rationality of sampling. The Pearson correlation coefficient between two biological replicates of six groups in this study had very high repeatability (i.e., all R^2^ ≥ 0.8804; Additional file 2: Figure S2).

**Table 1:** Characteristics of the reads from 12 duck skin transcriptomes

Sample	Raw bases (Gb)	Q20 value (%)	GC content (%)	Raw reads	Clean reads	Total mapped reads	Unique mapped reads^a^
EP1	4.23	97.41	52	47,016,964	45,760,796	33,808,649 (73.88%)	32,354,205 (70.70%)
EP2	4.25	97.47	53	47,310,314	46,088,812	33,990,213 (73.75%)	32,496,607 (70.51%)
EF1	4.00	96.92	48	44,475,234	42,914,954	33,946,076 (79.10%)	32,667,856 (76.12%)
EF2	4.31	96.78	51	47,959,362	45,914,254	35,291,893 (76.86%)	33,725,136 (73.45%)
MP1	4.30	97.59	55	47,812,176	46,738,350	33,041,341 (70.69%)	29,841,803 (63.85%)
MP2	4.36	97.58	54	48,498,554	47,459,658	32,965,478 (69.46%)	28,966,738 (61.03%)
MF1	4.36	97.49	52	48,448,010	47,364,954	35,355,911 (74.65%)	32,808,228 (69.27%)
MF2	4.28	97.68	51	47,560,598	45,252,442	34,196,920 (75.57%)	31,667,123 (69.98%)
LP1	4.35	97.13	49	48,414,006	46,679,934	37,128,381 (79.54%)	35,833,060 (76.76%)
LP2	4.35	97.21	48	48,369,596	46,514,142	37,313,860 (80.22%)	35,996,537 (77.39%)
LF1	4.34	97.16	48	48,217,072	46,385,418	37,094,213 (79.97%)	35,737,770 (77.05%)
LF2	4.35	97.07	48	48,378,418	46,602,248	37,258,174 (79.95%)	35,878,071 (76.99%)

^a^Unique mapped reads, reads that matched the reference genome in only one position

### Pairwise differential expression analyses and validation of Solexa sequencing data

**Fig. 1:**
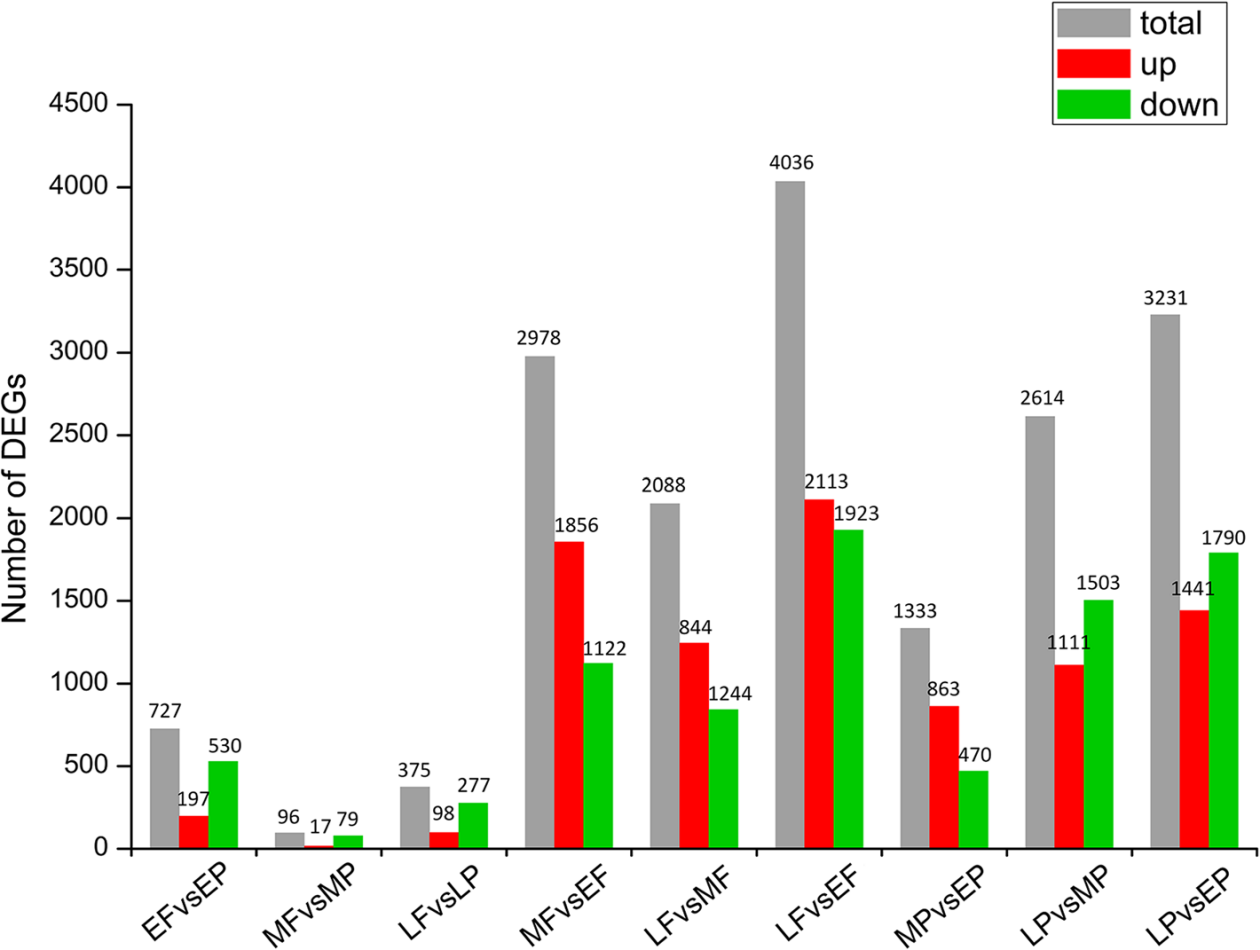
The number of differentially expressed genes between the comparison libraries. Total DEGs (grey), up-regulated genes (red), and down-regulated genes were presented by histogram

In this study, we used DEGseq to screen the differentially expressed genes (DEGs) to determine the differences in different feather types from different body parts or developmental stages. In the plumulaceous feather development, a total of 4756 genes were found to be DEGs; of these, 1333, 2614, 3231 genes were obtained in the comparison of MP *versus* EP, LP *versus* MP; LP *versus* EP, respectively. While in the process of the flight feather development, transcriptomic analyses identified a total of 5823 DEGs; of these, 2978, 2088, 4036 genes were screened in the comparison of MF *versus* EF, LF *versus* MF, LF *versus* EF, respectively. Transcriptomic analyses identified a total of 727, 96, 375 DEGs over 2-fold change that are possibly related to the differentiation of downy feather and flight feather in the comparisons of EF *versus* EP, MF *versus* MP, LF *versus* LP, respectively (Fig. 1). Ten DEGs were randomly selected for qRT-PCR quantification from the same RNA sample of MF and MP for the purpose of validating whether our sequencing and analysis were reliable. In general, high linear correlations and Pearson correlation coefficient of fold-change (FC) values from the two methods indicated that our transcriptome sequencing was reliable and we can make reasonable deductions from the gene expression values generated from RNAseq (Additional file 3: Figure S3).

### Functional genes and molecular events accounting for the development and morphogenesis of plumulaceous feather

The downy feathers of ducks are widely used as the filling materials of the insulation clothing. It is of great significance to study the morphogenesis and development of the plumulaceous feathers and identify the candidate genes participated in the process of plumulaceous production. In this part, we did some further analyses about three abdomen skin and follicle transcriptomes at different physiological stages, including series-cluster analysis, functional annotation, gene-act-network and gene co-expression analysis.

#### Series-cluster analysis and functional annotation of the clusters

**Fig. 2:**
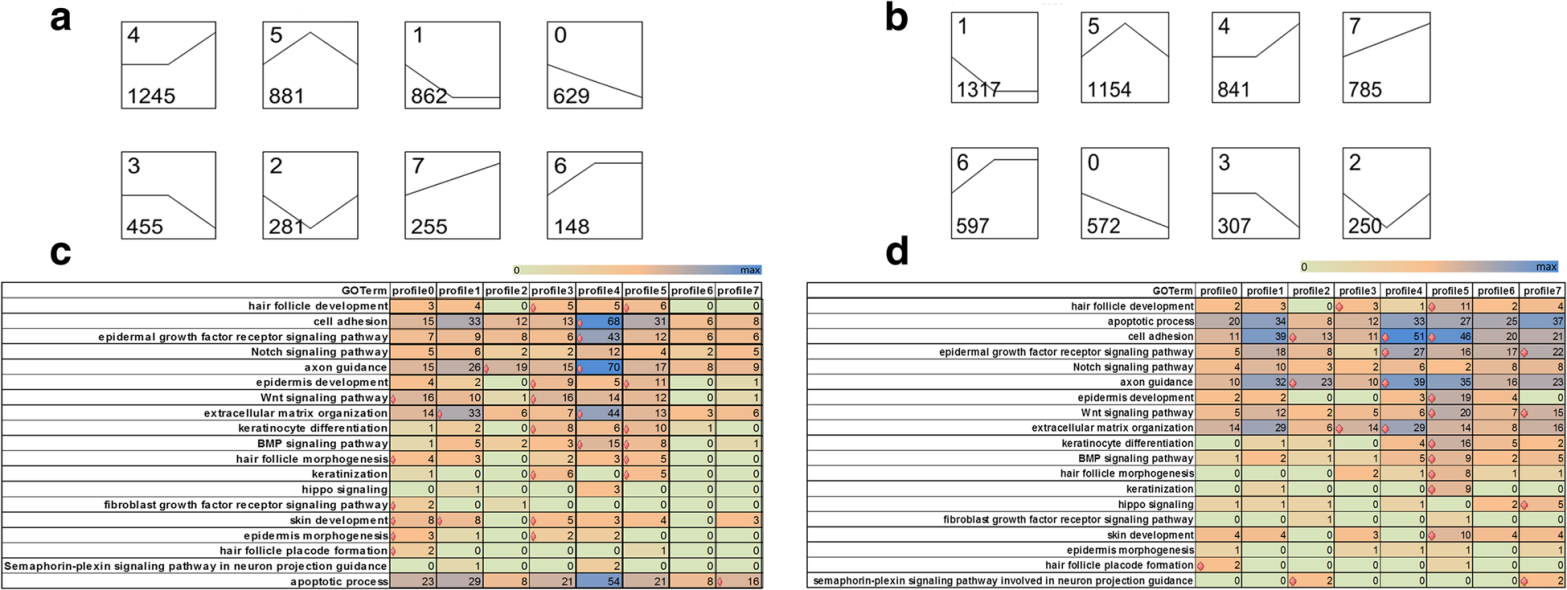
K-means clustering for DEGs in the P libraries or F libraries and GO functional enrichment analyses within clusters of two libraries. **a** K-means clustering for DEGs in the P libraries; **b** K-means clustering for DEGs in the F libraries; **c** Significant GO terms of DEGs in the P libraries associated with feather growth within 8 clusters; **d** Significant GO terms of DEGs in the F libraries associated with feather growth within 8 clusters. In the C and D, the x-axis is the type of cluster, y-axis is GO term associated with the feather development, the numbers in the rectangle indicates the number of DEGs participated in the GO term, diamond indicates enriched categories according to Fisher’s test (*p*-value ≤0.05)

The expression patterns not only indicate the diverse and complex interactions among genes, but also suggest that genes with similar expression patterns may have similar functions in the feather molting and growth. In the plumulaceous feather libraries (P libraries), a total of 4756 genes were found to be DE (Additional file 4: Figure S4). Then 8 series-clusters were obtained based on the 4756 DEGs (Fig. 2a; Additional file 5: Table S1). Each gene cluster exhibited a distinctive expression pattern. Largest group of P libraries is cluster 4 with 1245 (26.2%) genes, which maintained a relative stable expression from stage 1 to stage 2, and then rose at stage 3. GO enrichment analyses about 8 clusters were conducted for further understanding the gene expression pattern associated with the feather growth and development. Cluster 4 contained the largest number of DEGs that participate in the biological process of feather growth and development; these genes were associated with enriched GO terms including cell adhesion, epidermal growth factor receptor signaling pathway, axon guidance, extracellular matrix organization and BMP signaling pathway. Seven GO terms were enriched in cluster 3, including hair follicle development, epidermis development, WNT signaling pathway, keratinocyte differentiation, keratinization, skin development and epidermis morphogenesis. Meanwhile, no GO term that is associated with feather growth and development was enriched in cluster 6 (Fig. 2c).

#### GO and pathway analysis of DEGs in P libraries

The significant enriched GO terms (*p*-value < 0.05) involved in the plumulaceous feather development were determined from the DEGs of MPvsEP, LPvsMP and LPvsEP (See for full list of GO terms in Table 2). Keratinization, extracellular matrix organization, cell adhesion, hair follicle development, keratinocyte differentiation and hair follicle morphogenesis were all significantly enriched in the DEGs of MP/EP, LP/MP and LP/EP. Different KEGG pathways accounting for plumulaceous feather development were determined in 3 different comparison libraries (See for full list of KEGG pathway in Additional file 6: Table S2). These pathways are suggested to be important during the morphogenesis and growth of the plumulaceous feather in duck, including Adherens junction, MAPK signaling pathway, Hippo signaling pathway, Hedgehog signaling pathway, cell adhesion molecules (CAMs), ECM-receptor interaction, TNF signaling pathway, Jak-STAT signaling pathway, and Wnt signaling pathway.

**Table 2:** GO functional enrichment analysis related with feather development of the DEGs of MPvsEP, LPvsMP and LPvsEP

Tissue comparison	GO Term	Enricnment score	P-value
MPvsEP	extracellular matrix organization	1.71	0.0026
	keratinocyte differentiation	2.64	0.0064
	morphogenesis of a branching epithelium	12.48	0.0069
	cell adhesion	1.45	0.0087
	keratinization	3.29	0.0292
	neurotrophin signaling pathway	12.48	0.0299
	negative regulation of hair follicle development	12.48	0.0299
	hair follicle development	2.38	0.0302
	hair follicle morphogenesis	2.50	0.0477
	cellular response to epidermal growth factor stimulus	2.50	0.0478
LPvsMP	cell adhesion	1.57	1.99E-05
	keratinocyte differentiation	2.81	8.43E-05
	SMAD protein signal transduction	2.72	9.22E-05
	epidermis development	2.47	0.0001
	cell-matrix adhesion	2.17	0.0003
	extracellular matrix organization	1.56	0.0009
	epithelial to mesenchymal transition	2.75	0.0011
	hair follicle development	2.61	0.0012
	keratinization	3.53	0.0018
	integrin-mediated signaling pathway	1.91	0.0038
	hair follicle morphogenesis	2.64	0.0051
	BMP signaling pathway	1.97	0.0077
	Wnt signaling pathway	1.54	0.0098
	morphogenesis of an epithelium	2.44	0.0113
	epithelial cell differentiation	1.65	0.0380
	cell-cell adhesion mediated by integrin	4.06	0.0410
	cellular response to transforming growth factor beta stimulus	1.84	0.0456
LPvsEP	extracellular matrix organization	1.68	2.68E-05
	cell-matrix adhesion	1.89	0.0018
	cell adhesion	1.36	0.0021
	morphogenesis of an epithelium	2.51	0.0048
	keratinocyte differentiation	2.20	0.0069
	integrin-mediated signaling pathway	1.75	0.0079
	regulation of Notch signaling pathway	2.86	0.0208
	Wnt signaling pathway	1.42	0.0242
	keratinization	2.37	0.0330
	hair follicle morphogenesis	2.01	0.0373
	BMP signaling pathway	1.62	0.0417
	hair follicle development	1.79	0.0432

A global pathway net was constructed based on the significant pathways to illustrate the key pathways in the process of plumulaceous feather development (Additional file 7: Figure S5). Adherens junction and Wnt signaling pathway were considered to be the most important node in the net because the component exchanges with other pathways were strongly dependent on its existence. Adherens junction, one of the cell junctions, exists in the different types of epithelial cell (including hair follicle epithelium) [18]. In the process of hair follicle morphogenesis and development, Wnt pathway plays an essential role during hair follicle induction and is considered to be master regulator during hair follicle morphogenesis [19].

#### Gene-act-network

**Fig. 3:**
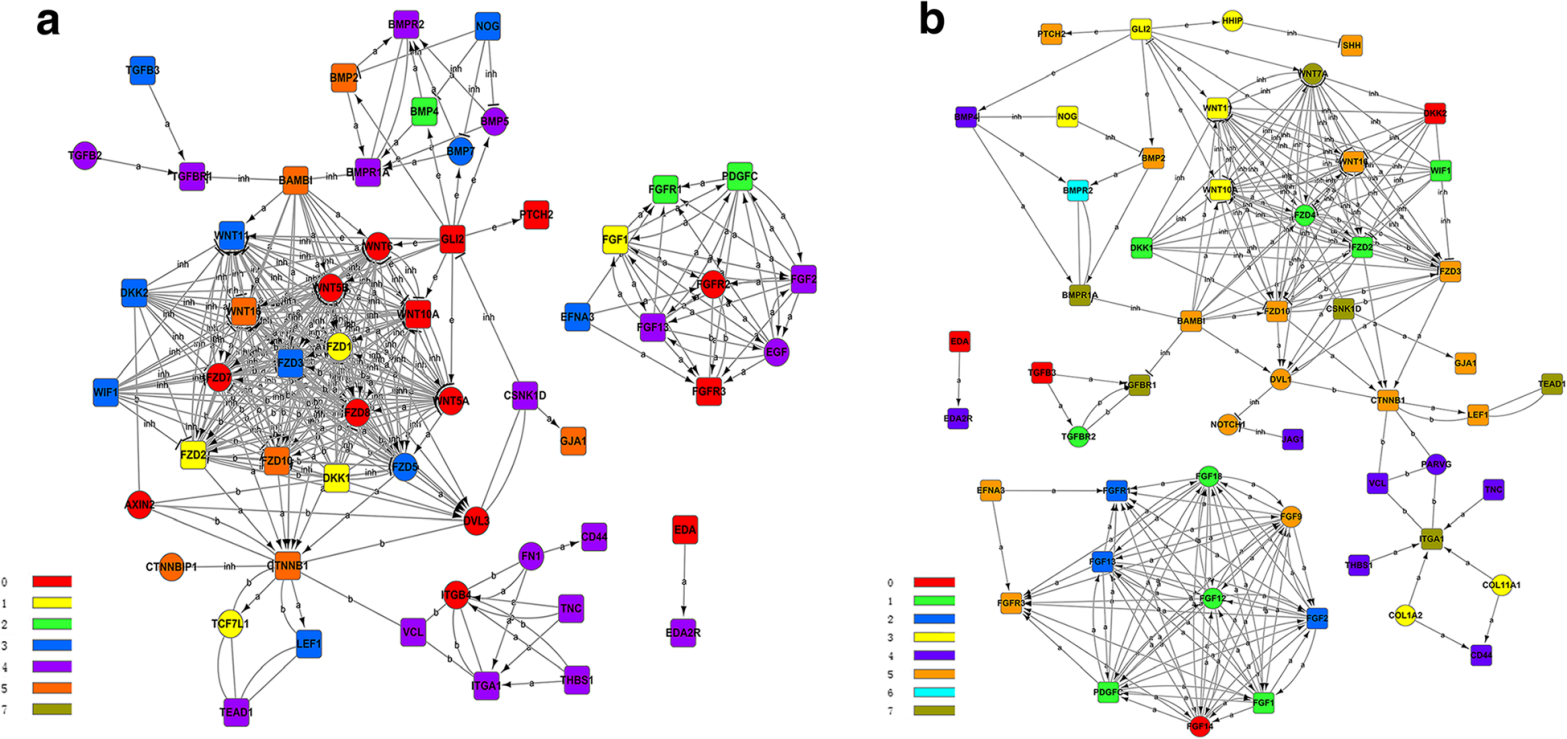
Gene-act-network analyses of candidate genes involved in the feather development. **a** Gene-act-network of candidate genes from abdomen skin and follicle transcriptomes; **b** Gene-act-network of candidate genes from wing skin and follicle transcriptomes; Different colours of genes indicate they belong to different clusters

After functional analysis, it is important to explore the relationships among the DEGs involved in the aforementioned biological processes. In the gene interaction network, a wide variety of Wnt family (*WNT5A*, *WNT5B*, *WNT6*, *WNT10A*, *WNT11*, *WNT16*), and its receptors of frizzled protein (*FZD1*, *FZD2*, *FZD3*, *FZD5*, *FZD7*, *FZD8*, *FZD10*) and DKK protein (*DKK1*, *DKK2*), *CTNNB1*, *AXIN2*, *BAMBI*, *WIF1*, *TCF7L1*, *LEF1* were involved in the key pathways that previously mentioned including Wnt signaling pathway and adherens junction (Fig. 3a). *WNT5A* is confirmed to express almost in the mesenchymal and epidermal cell before the feather bud formation, and is a target of SHH in hair follicle morphogenesis [20]. The quantity of downy feather is mainly affected by the follicles development in birds, *WNT6* plays a key role in follicular development as an intercellular signaling molecule, and its polymorphism is also related to the follicle development in Chinese indigenous Wanxi-white goose [21]. Common polymorphisms in *WNT10A* have effects on the morphology of ectodermal appendages, including tooth and hair [22]. Frizzled proteins, a G protein-coupled receptor family, mainly have functions in three distinct signaling pathways (canonical Wnt/β-catenin pathway, Wnt/calcium pathway and planar cell polarity (PCP) pathway) [23]. In this part, seven of ten Frizzled proteins have expressed differently in the three stages of down feather development. Furthermore, we found that *FZD1*, *FZD3*, *FZD7* and *FZD8* have complex interactive relationships with other genes through gene-act-network analysis. Whether Frizzled proteins are related to the growth and development of downy feather is an interesting question that will be explored in future study. Dkk2/Frzb, an inhibitor for Wnt signaling, could regulate the feather regeneration [24]. In Fig. 3a, *CTNNB1*, which encode β-catenin, is located in the center of gene-act-network. In the morphogenesis of feather, β-catenin plays a significant role in the formation of feather bud [25].

#### Gene-co-expression

**Fig. 4:**
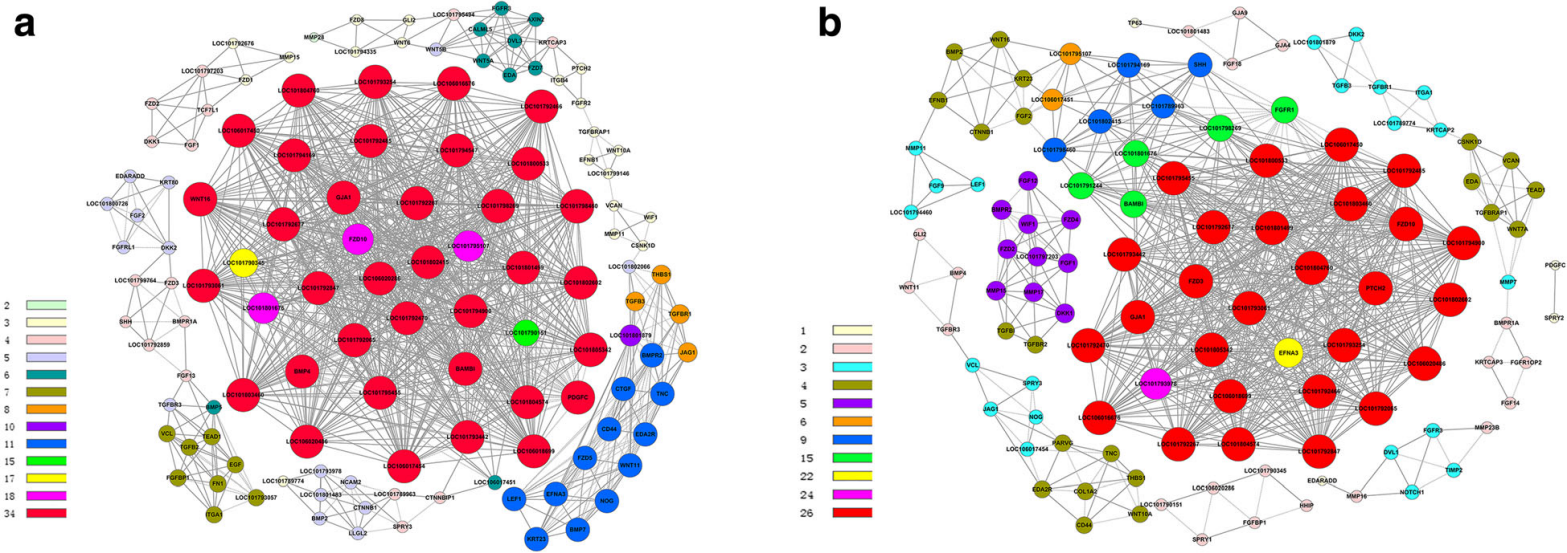
Co-expression analyses of candidate genes involved in the feather development. **a** Co-expression analysis of candidate genes from P libraries; **b** Co-expression analysis of candidate genes from F libraries. Different colours of genes indicate they belong to different clusters

Alternatively, we performed the gene co-expression net-work analysis of DEGs in the P libraries (Fig. 4a, Table 3). There are 35 genes with highest K-core values (K-core = 34) in the gene co-expression analysis, which possibly are the key genes involved in the plumulaceous feather; of these, 30 of 35 are keratins or keratin-associated proteins (including 4 α-keratins, 2 KAPs, 1 β-keratin and 23 feather keratins), thus indicating that feather keratins play significant roles in the growth and development of plumulaceous feather. In addition, five other genes also have highest K-cores in this part, including *BMP4*, *GJA1*, *PDGFC*, *WNT16* and *BAMBI*. *PDGFC* (platelet derived growth factor C) could enhance the feather growth and its receptor expressed in feather collar [26]. Gene co-expression analysis could find out the key genes that may regulate the growth and development of downy feather, which enriched the comprehensive understanding of plumulaceous growth from the aspect of omics.

**Table 3:** 35 genes with highest K-core value of candidate genes involved in the development of plumulaceous feathers

GeneID	Description	Degree	K-core
*BMP4*	Bone morphogenetic protein 4	40	34
*GJA1*	Gap junction protein	39	34
*LOC101792485*	Feather keratin 1	39	34
*LOC101794169*	Keratin, type I cytoskeletal 19	39	34
*LOC101794547*	Keratin, type I cytoskeletal 9-like	39	34
*LOC101798269*	Feather keratin 1	39	34
*LOC101798460*	Feather keratin 1-like	39	34
*LOC101802415*	Feather keratin 1	39	34
*LOC106020286*	Keratin-associated protein 19–2-like	39	34
*PDGFC*	Platelet-derived growth factor C	39	34
*WNT16*	Protein Wnt-16	39	34
*BAMBI*	BMP and activin membrane-bound inhibitor homolog	38	34
*LOC101792677*	Feather beta keratin	38	34
*LOC101803460*	Feather keratin 1	38	34
*LOC101793061*	Feather keratin 1	37	34
*LOC101801499*	Feather beta keratin	37	34
*LOC106020486*	Feather beta keratin-like	36	34
*LOC101792065*	Feather keratin Cos2–3-like	34	34
*LOC101792267*	Feather keratin Cos1–2	34	34
*LOC101792466*	Feather keratin Cos1–2-like	34	34
*LOC101792470*	Feather keratin 2	34	34
*LOC101792847*	Feather beta keratin	34	34
*LOC101793254*	Fether keratin 1-like	34	34
*LOC101793442*	Feather keratin 1	34	34
*LOC101794900*	Keratin, type I cytoskeletal 15	34	34
*LOC101795455*	Feather beta keratin	34	34
*LOC101800533*	Keratin, type II cuticular Hb4	34	34
*LOC101802602*	Feather keratin Cos2–3	34	34
*LOC101804574*	Feather keratin Cos1–1/Cos1–3/Cos2–1	34	34
*LOC101804760*	Feather keratin Cos2–3-like	34	34
*LOC101805342*	Feather keratin Cos1–1/Cos1–3/Cos2–1-like	34	34
*LOC106016676*	Feather keratin Cos1–1/Cos1–3/Cos2–1	34	34
*LOC106017450*	Keratin-associated protein 5–5-like	34	34
*LOC106017454*	Feather keratin 4-like	34	34
*LOC106018699*	Beta-keratin-related protein-like	34	34

### Functional genes and molecular events accounting for the development and morphogenesis of flight feather

The flight feather is an unparalleled structure, which has two contradictory characters; flight feather consists of light material and it could withstand strong air resistance when birds fly. Flight feather is the best research material for evolutionary biology and developmental biology. In this part, same methods were used to clarify the functional genes and molecular events associated with the development and morphogenesis of flight feather.

#### Series-cluster analysis and functional annotation of the clusters

To better understand the significant expression pattern related to the growth and development of flight feather, same analysis was conducted based on the 5823 DEGs (F libraries) and 8 clusters were also achieved (Additional file 8: Table S3). Largest group of F libraries is cluster 1 with 1317 (22.6%) genes; the expression levels in this cluster declined gradually from stage 1 to stage 2, and maintained a relative stable expression from stage 2 to stage 3 (Fig. 2b). However, there is no GO term concerned with feather morphogenesis that was enriched in cluster 1 and 6. While cluster 5 contained the largest number of DEGs that participate in the biological process of feather growth and development, as well as the most significant GO terms; these GO terms included hair follicle development, cell adhesion, epidermis development, WNT signaling pathway, keratinocyte differentiation, BMP signaling pathway, hair follicle morphogenesis, keratinization and skin development (Fig. 2d).

#### GO and pathway analysis of DEGs in F libraries

The significant enriched GO terms were identified through different comparisons (See for full list of GO terms in Table 4). Intracellular signal transduction was significantly enriched in the DEGs of MF/EF, LF/MF, LF/EF. Likewise, KEGG pathway related to flight feather development were also recognized (See for full list of KEGG pathway in Additional file 9: Table S4), including MAPK signaling pathway, VEGF signaling pathway, Jak-STAT signaling pathway, Focal adhesion, ECM-receptor interaction, TNF signaling pathway, cell adhesion molecules (CAMs), adherens junction and NF-kappa B signaling pathway.

**Table 4:** GO functional enrichment analysis related with feather development of the DEGs of MFvsEF, LFvsMF and LFvsEF

Tissue comparison	GO Term	Enricnment score	P-value
MFvsEF	keratinocyte differentiation	2.38	0.0008
	anterior/posterior pattern specification	1.71	0.0078
	epidermis development	1.80	0.0138
	hair follicle development	2.05	0.0146
	intracellular signal transduction	1.27	0.0204
	keratinization	2.55	0.0230
LFvsMF	cell adhesion	1.78	5.01E-07
	extracellular matrix organization	1.87	1.87E-05
	integrin-mediated signaling pathway	2.41	0.0002
	cell-matrix adhesion	2.21	0.0008
	multicellular organismal development	1.36	0.0010
	axon guidance	1.47	0.0010
	keratinization	3.77	0.0026
	keratinocyte differentiation	2.45	0.0029
	epidermis development	2.08	0.0072
	epithelial to mesenchymal transition	2.30	0.0176
	hair follicle morphogenesis	2.39	0.0256
	intracellular signal transduction	1.28	0.0403
	anatomical structure formation involved in morphogenesis	2.28	0.0420
LFvsEF	extracellular matrix organization	1.44	0.0018
	integrin-mediated signaling pathway	1.73	0.0049
	cell-matrix adhesion	1.61	0.0130
	intracellular signal transduction	1.21	0.0331

#### Gene-act-network

Gene-act-network was constructed to ascertain the relations among the DEGs in F libraries (Fig. 3b). In this gene interaction network, we could find that few DEGs belong to BMP families were screened in F libraries, including *BMP2*, *BMP4*, *BMPR2* and *BMPR1A*. *BMP2* is expressed in the posterior region of the feather buds. BMP and SHH pathways are involved in regulating the formation and balance among the rachis and barbs. A wide variety of WNT family (*WNT7A*, *WNT10A*, *WNT11*, *WNT16*, *FZD2*), and its receptors of frizzled protein (*FZD2*, *FZD3*, *FZD4*, *FZD10*) and DKK protein (*DKK1*, *DKK2*), as well as DEGs belong to FGF family (*FGF1*, *FGF2*, *FGFR1*, *FGFR3*, *FGF9*, *FGF12*, *FGF13*, *FGF14*, *FGF18*) were determined in the F libraries. Some particular DEGs (such as *HHIP* and *DVL1*) were identified only in F libraries comparing with P libraries. *HHIP* interact with hedgehog family to regulate the hedgehog signaling of several cell types [27].

#### Gene-co-expression

Gene co-expression net-work analysis of DEGs of F libraries was constructed (Fig. 4b, Table 5). Core genes with the highest degrees connect with most adjacent genes in the network and are frequently identified as key indicators. There are 29 genes with highest k-core values in the gene co-expression analysis; of these, 24 are keratins or keratin-associated proteins, thus indicating that feather keratins also take important roles in the growth and development of flight feather, as well as in the plumulaceous feather. In addition, five other genes also have highest K-cores in this part, including *GJA1*, *FZD3*, *FZD10*, *PICH2* and *EFNA3*. Repression of Hh (Hedgehog) signaling through a dynamic *PTCH1* and *PTCH2* regulatory network is a crucial event in lineage fate determination in the skin [28]. *FZD3 and FZD10* encode Wnt receptors, and the research has shown that *FZD*10 is also expressed in the feather bud [29]. In addition, *FZD3* and *FZD10* also have relationships with several the other genes in the gene-act-network; hence we speculate that *FZD3* and *FZD10* may have certain effect on the growth and development of the flight feather.

**Table 5:** 29 genes with highest K-core value of candidate genes involved in the development of flight feather

GeneID	Description	Degree	K-core
*LOC101794900*	keratin, type I cytoskeletal 15	34	26
*PTCH2*	Protein patched homolog 2	34	26
*FZD10*	Frizzled-10	33	26
*LOC101792485*	feather keratin 1	33	26
*LOC101792677*	feather beta keratin	33	26
*LOC101792847*	feather beta keratin	33	26
*LOC101801499*	feather beta keratin	33	26
*LOC101803460*	feather keratin 1	33	26
*LOC106020486*	feather beta keratin-like	33	26
*FZD3*	Frizzled-3	32	26
*GJA1*	Gap junction alpha-1 protein	32	26
*LOC101800533*	keratin, type II cuticular Hb4	32	26
*LOC101792065*	feather keratin Cos2–3-like	28	26
*LOC101792267*	feather keratin Cos1–2	28	26
*LOC101792466*	feather keratin Cos1–2-like	28	26
*LOC101792470*	feather keratin 2	28	26
*LOC101793061*	feather keratin 1	28	26
*LOC101793254*	feather keratin 1-like	28	26
*LOC101793442*	feather keratin 1	28	26
*LOC101795455*	feather beta keratin	28	26
*LOC101802602*	feather keratin Cos2–3	28	26
*LOC101804574*	feather keratin Cos1–1/Cos1–3/Cos2–1	28	26
*LOC101804760*	feather keratin Cos2–3-like	28	26
*LOC101805342*	feather keratin Cos1–1/Cos1–3/Cos2–1-like	28	26
*LOC106016676*	feather keratin Cos1–1/Cos1–3/Cos2–1	28	26
*LOC106017450*	keratin-associated protein 5–5-like	28	26
*LOC106018699*	beta-keratin-related protein-like	28	26
*LOC101793978*	keratin, type I cytoskeletal 14-like	25	24
*EFNA3*	Ephrin-A3	22	22

## Discussion

### Developmental transcriptomes comparisons between plumulaceous feather and flight feather

The developmental transcriptomes of two different body parts were compared to do further research of the growth and development differences between plumulaceous feather and flight feather. Many biological processes were both enriched in the DEGs from P and F libraries. Cell adhesion molecules may regulate feather morphogenesis by straining cell motion and forming borders [30]. The studies on the mouse have shown that inhibiting Jak-STAT pathway makes hair of rapid growth [31]. ECM-receptor interaction plays a fundamental role in the morphogenesis of tissues and organs, and their main functions are the maintenance the structure of organs and functional homeostasis, and in the control of the gene expression [32]. The studies on Cashmere goat showed that high expression of ECM and cell surface proteins was essential for the rapid growth of hair follicles during the anagen phase [33].

However, in the P developmental transcriptomes, 289 GO terms are enriched in the DEGs of LPvsEP, such as SMAD protein signal transduction (GO: 0060395), which is not an enriched GO term in F libraries. SMADs are a group of signaling mediators and antagonists of the transforming growth factor-beta (TGF-beta) superfamily, as well as responding to Activin and BMPs, which play important roles in skin development. *SMAD4* affects hair follicle differentiation through regulating BMP signaling. *SMAD7* significantly has impact on the hair follicle development and differentiation by blocking the TGFbeta/Activin/BMP pathway and by inhibiting WNT/beta-catenin signaling [34]. NF-kappa B signaling pathway is significantly enriched only in the F libraries. Activation of the NF-kappaB pathway by the Edar and Edaradd is required for the development of hair follilces [35]. And NF-kappa B activation is essential for induced SHH and cyclin D1 expression and subsequent hair placode down growth [36]. In addition, there are lots of DEGs between P and F libraries, especially the DEGs from BMP, WNT and FGF families. The differences of DEGs are not only the numbers and varieties, even if the same DEGs possibly have different expression trends in P and F libraries. For example, *CRABP1* has been detected belonging to the profile 3 and 1 in P library and F library, respectively. The differential levels of *CRABP1* and some other genes over different body regions and time would shape the anisotropic RA landscape, in order to introduce a new dimension of vane shape variations [37]. Therefore, we speculated that the existing genes construct the different gene expression networks in the skin and follicles of duck different body parts and developmental stages, so as to further differentiate into diverse feathers.

### Functional genes involved in the differentiation of plumulaceous feather and flight feather

**Fig. 5:**
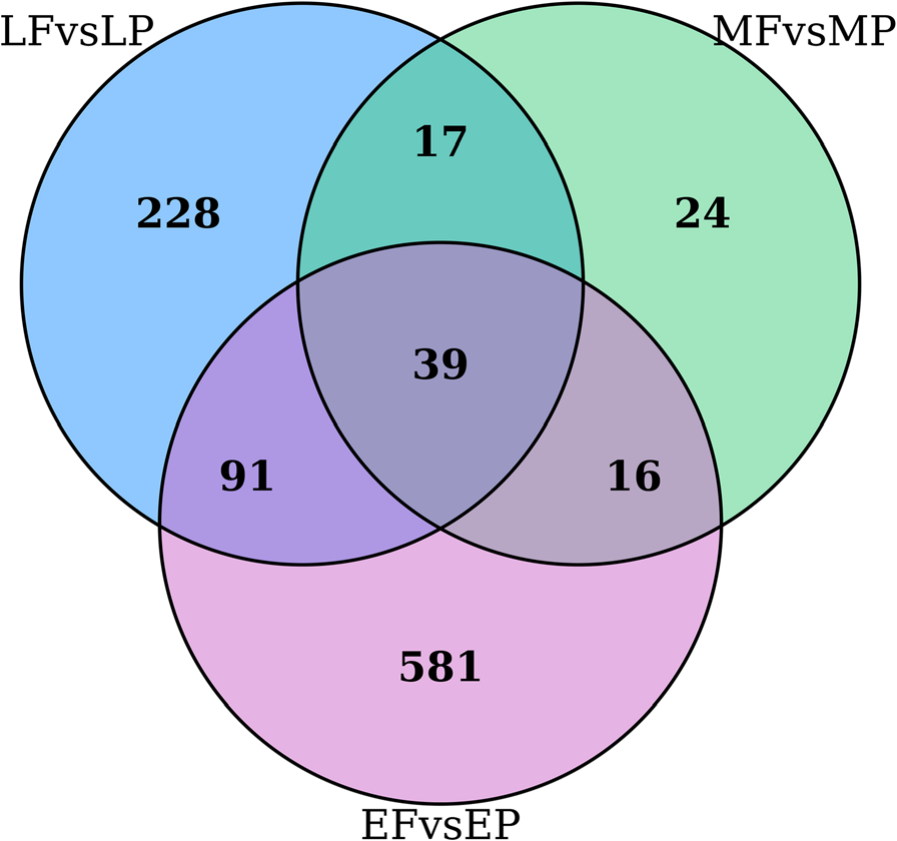
A venn diagram of the number of unique and shared DEGs in the comparisons of EF/EP, MF/MP and LF/LP

After we thoroughly investigated the DEGs of abdomen and wing skin transcriptomes at the same physiological stages, two key genes (*MGP* and *PITX2*) were screened in the two of the three comparisons of EFvsEP, MFvsMP and LFvsLP. The interaction of matrix GLA protein (*MGP*, an inhibitory morphogen) and *BMP4* (an activating morphogen) are suggested to be important for vascular branching [38], but has not been reported to play any roles in feather morphogenesis. *MGP* is likely to facilitate rachis and barb branching in chicken feathers. The transcription factor *PITX2*, is a key factor in left-right asymmetry during the process of vertebrate development [39]. In chick embryos, early asymmetric expression of *PITX2* leads to asymmetric ovarian development [40]. Another intriguing finding of this work is the identification of a total of 39 DEGs were shared across all three comparisons (Fig. 5); of these, 38 genes have consistent expression pattern in abdomen or wing skin transcriptome (Table 6). It is very interesting to find that *HOXD10*, *HOXD11* and *FMOD* are all up-regulated in the EF, MF and LF. While the other 35 genes are up-regulated in the EP, MP and LP, including eight genes belonging to *HOX* cluster (*HOXB2*, *HOXB3*, *HOXB4*, *HOXB5*, *HOXB6*, *HOXB8*, *HOXC9* and *HOXC10)*, one transcription factor *TBX4*, two feather keratin (*LOC101804574 and LOC106016676)*, as well as other genes.

**Table 6:** 39 shared differentially expressed genes in EF/EP, MF/MP and LF/LP pairwise analysis

GeneID	FC_EF/EP_	FC_MF/MP_	FC_LF/LP_	Putative description
*HOXD10*	4.81	3.17	4.90	Homeobox protein Hox-D10
*HOXD11*	392.92	53.51	1290.81	Homeobox protein Hox-D11
*FMOD*	4.65	3.48	2.60	Fibromodulin
*HOXB2*	−5.54	−5.92	−4.16	Homeobox protein Hox-B2
*HOXB3*	−4.72	−17.62	−12.03	Homeobox protein Hox-B3
*HOXB4*	−5.52	−7.53	−7.71	Homeobox protein Hox-B4
*HOXB5*	−9.25	−12.57	−20.66	Homeobox protein Hox-B5
*HOXB6*	−3.38	−6.01	−7.74	Homeobox protein Hox-B6
*HOXB8*	−3.64	−30.29	−7.40	Homeobox protein Hox-B8
*HOXB9*	−14.69	− 29.81	− 25.00	Homeobox protein Hox-B9
*HOXC10*	−27.28	−40.96	−15.22	Homeobox protein Hox-C10
*LOC101793166*	− 129.84	−18.66	−55.71	Mid1-interacting protein 1-B-like
*TMEM255B*	−11.23	−5.52	−8.57	Transmembrane protein 255B
*TBX4*	−12.92	−19.21	−33.32	T-box transcription factor TBX4
*LOC101794524*	−4.53	−5.86	−3.67	Desmin
*SSTR2*	−29.11	−14.56	− 237.38	Somatostatin receptor 2
*SLC25A4*	−2.38	−3.47	−2.96	Solute carrier family 25 member 4
*LOC101802284*	−3.35	−2.95	−4.60	Uncharacterized LOC101802284
*LOC106017141*	−10.84	−6.88	−23.75	Chromosome unknown open reading frame, human C12orf75
*LOC101804574*	−8.64	−2.62	− 1361.13	Feather keratin Cos1–1/Cos1–3/Cos2–1
*P2RX1*	−6.61	−3.41	− 2.82	Purinergic receptor P2X, ligand-gated ion channel, 1
*HSPH1*	−4.12	−2.61	−2.31	Heat shock 105 kDa/110 kDa protein 1
*IYD*	−10.11	−6.85	− 64.88	Iodotyrosine deiodinase
*ACTG2*	−4.18	− 4.60	−2.59	Actin, gamma 2, smooth muscle, enteric
*LOC106019157*	−4.05	−5.96	−12.19	Uncharacterized LOC106019157
*LOC101804390*	−3.87	−3.42	−2.23	Glycogen phosphorylase, brain form
*CMYA5*	−10.21	−2.84	−4.72	Cardiomyopathy associated 5
*MYL9*	−4.53	−5.24	−3.02	Myosin, light chain 9, regulatory
*FHL2*	−6.06	−4.51	−4.07	Four and a half LIM domains 2
*DES*	−4.49	−6.19	−3.66	Desmin
*ACACB*	−10.59	−10.10	−23.82	Acetyl-CoA carboxylase beta
*PPP1R3C*	−2.90	−3.17	−20.93	Protein phosphatase 1, regulatory subunit 3C
*EPHX4*	−4.03	−5.89	−10.85	Epoxide hydrolase 4
*LOC101790040*	−5.23	−4.74	−4.47	Coronin-6
*G0S2*	−9.38	−4.37	−3.94	G0/G1 switch 2
*ABHD6*	−10.56	−14.66	−3.47	Abhydrolase domain containing 6
*CUTA*	−3.40	−4.31	−2.67	CutA divalent cation tolerance homolog
*LOC106016676*	−9.62	−2.57	− 118.07	Feather keratin Cos1–1/Cos1–3/Cos2–1
*ACTN2*	3.15	−3.84	−42.94	Actinin alpha 2

In the growth and development of hair follicle, HOX genes have been confirmed to express in the mice hair follicle among different developmental periods, and multiple members of HOX genes have been considered to play vital roles in the hair follicle. The spatial expression patterns of HOXA and HOXB cluster were correlated with morphological subdivision of the digestive tract along the anteroposterior axis [41]. *HOX13* is the first homeobox gene shown to have overt phenotypic effects on hair development. Abnormal expression of *HOXC13* could cause the defect of hair growth to generate hairless mice [42]. *HOXC8* was the only candidate gene examined with a strikingly altered expression pattern, which may directly influence the development of feathers, especially in terms of the morphology of the cranial feathers and thus cause the Crest phenotype in chicken [16]. *HOXB5* regulates mesodermal-epithelial crosstalk during development, and loss of its function leads to the downregulation of previously identified downstream targets of Wnt2/2b signaling, including *LEF1*, *AXIN2*, and *BMP4* [43]. In general, different HOX clusters have differentially expression levels between plumulaceous feather and flight feather at all three physiological stages, and they possibly participated in the formation of diverse feathers.

## Conclusions

In conclusion, we provide a new insight into transcriptional profiles of two feather types development at three stages using a genome-wide deep sequencing method. The morphogenesis and development of avian feathers are possibly regulated by a complicated process, including a series signal transduction molecules, growth factors and transcription factors, several types of cell connections, abundant members from HOX and feather keratin families. Furthermore, this study of feather transcriptional profiles is helpful in understanding the differences between the genetic mechanisms in plumulaceous and flight feathers, and for future work exploring genetic basis of feather divergence and development.

## Methods

### Ethics statement

This study was carried out in strict accordance with the recommendations of the Regulations for the Administration of Affairs Concerning Experimental Animals (Ministry of Science and Technology, China, revised in June 2004). Housing and caring of Cherry Valley ducks and collection of skin samples for use in the described experiments were conducted following the approved protocol of the Institutional Animal Care and Use Committees of college of Life Sciences, Shaanxi Normal University. All ducks in this study were given continuous access to a standard commercial feed ration and water. All surgery was performed under combination anesthesia, and all efforts were made to minimize suffering of animals.

### Sample rearing, sampling of and RNA extraction from skin tissues

The samples of Cherry Valley ducks were selected from Sichuan Xinmianying Farming Corporation. And we maintained all of these individuals in the same raising environment (fed with pellet feed and green vegetables) at the Institute of Zoology, Shaanxi Normal University, Xi’an, Shaanxi Province, China, in 2014. Male ducks at three different physiological stages (3-day-old, 27-day-old and 6-month-old) were selected and divided into early growth phase (E), middle growth phase (M) and late growth phase (L) groups. First, the feathers born in the abdomen and inner side of the wing at each stage were sheared and further shaved (we have not pulled out the whole feathers). Then the ducks were locally anaesthetized with pentobarbital sodium (3%, 1 ml/kg) through intraperitoneal injection to minimize the animal suffering. A piece of skin (1 cm in diameter) owing feather follicle from the abdominal and wing side were collected with scissors and forceps and immediately washed with 10 mL PBS (PH7.2) and 0.5 mM EDTA. Each sample was then placed in RNAlater (Ambion) and stored at 4 °C for 24 h first and then at − 80 °C until RNA extraction.

RNAlysis was performed using TRIzol Reagent (Invitrogen, USA) followed by isolation and purification with the RNeasy kit (Qiagen, Germany). RNA degradation and contamination was assessed on 1% agarose gel electrophoresis. RNA purity and integrity were evaluated using the NanoPhotometer spectrophotometer (IMPLEN, CA, USA) and the Agilent 2100 Bioanalyzer (Agilent Technologies, Santa Clara, CA, USA), respectively.

### Library preparation and sequencing

Twelve samples with RNA integrity number (RIN) values above 7 were used for libraries construction. Sequencing libraries were generated using the IlluminaTruSeq™ RNA Sample Preparation Kit (Illumina, San Diego, USA) following the manufacturer’s recommendations. Briefly, Oligo(dT) magnetic beads were used to isolate Poly(A) mRNA from total RNA. Then purified mRNA was interrupted into fragments with divalent cations. Double-stranded cDNA was synthesized using random hexamer-primers, reverse transcriptase, DNA polymerase I and RNaseH by taking short fragments as templates. Subsequently, double-stranded cDNA was further subjected to end-repair and ligation with adapters. These modified products were purified and enriched with PCR to construct the final cDNA library. After test the quality of libraries, they were loaded onto the flow cell channels of an Illumina HiSeq™ 2000 platform and 90 bp pair-end reads were generated at BGI, Shenzhen, China.

### Sequence reads mapping and assembly

Be assembly, raw reads of fastq format were firstly filtered by removing reads containing adaptors, reads containing poly-N and low quality reads to obtain high-quality clean reads under following criteria: a. Reads contain 20% base quality lower than Q20; b. Reads length ≥ 50 bp; c. Trim N-end. At the same time, quality parameters of clean data including Q20 and GC-content were obtained. All the succeeding analyses were carried out using high quality clean reads. Clean reads were mapped to the Peking duck genome assembly BGI_duck_1.0 (GCA_000355885.1) by using TopHat2 [44] with following parameter (tophat -r 272 -a 10 -m 0 -i 31 -I 500000 --read-mismatches 3 --max-insertion-length 6 --max-deletion-length 6 --read-gap-length 7 --read-edit-dist 12 --b2-sensitive --solexa1.3-quals -p 8 --min-coverage-intron 31 --min-segment-intron 31).

### Quantification and identification of differentially expressed genes (DEGs)

The parameter FPKM (Fragments per kilobase of transcript per million mapped reads) was applied to quantify the gene expression levels by using BGI_duck_1.0 genome annotation gtf file. Gene expression calculated through FPKM could eliminate the influence of gene length and sequencing data on gene expression. HTseq [45] was used for count calculation and FPKM was calculated with NCBI gtf file through gene length annotation by domestic code. We used the calculated gene expression to compare the differences in gene expression between the skin transcriptome of abdomen and wing during three developmental stages. The DEGseq package was applied to filter the differentially expressed genes with a fold change > 2 or fold change < 0.5, and false discovery rate (FDR) < 0.05 [46, 47].

### Classification, annotation and co-expression of DEGs

Series cluster analysis was performed using STEM [48] to classify the differentially expressed genes in eight clusters based on the FPKM change tendency of genes in three developmental stages. Fisher’s exact test and the multiple comparison tests were used to calculate the significant levels of profiles [49, 50].

Gene ontology (GO) analysis was performed to facilitate elucidating the biological implications of unique genes in the significant or representative profiles, which helps us to find those GOs with more concrete function description in this study [51]. Generally, Fisher’s exact test was applied to idenfity the significant GO categories and FDR was used to correct the *p*-values. The threshod of significance was defined by p-value < 0.05 and FDR < 0.05. Within the significant category, the enrichment was given by: Enrichment = (n_f_/n)/(N_f_/N), where “N” was the number of BG genes (background-genes) which were achieved from the genome gtf file download from NCBI, “n” was the number of total DEGs or the genes we analyzed with GO annotation, “n_f_” and “N_f_” represent the DEGs and BG genes with the target GO-term annotation .

Pathway analysis was used to find out the significant pathway of the differential expressed genes according to the KEGG database. A Fisher exact test was used to find the significant enrichment pathway with the threshod of significance of p-value < 0.05 and FDR < 0.05, which helps us to find those more significant pathways in this study. The calculation method of enrichment value is similar with the GO enrichment.

Pathway-act-network analysis was performed to reveal the interactive network among the pathways with enriched DEGs based on the KEGG database, while graphical representations of the pathways were generated based on the Cytoscape software [52]. The core pathway which is related with the morphogenesis and development of feather could be identified by building the signaling pathway network.

Gene-act-net analysis was conducted to reveal the network of the DEGs based on the interactions among the genes, proteins and compounds included in the KEGG database. In the network, cycle nodes represent genes, and edges between two nodes represent interactions between genes.

Gene co-expression network analysis was performed to track the interactions among the DEGs, according to their dynamic expression changes in three developmental stages. Pearson correlation was applied to each pair of genes and the significantly correlated pairs were used to construct the network [53]. K-core scoring of a given gene, which indicates its hub or nodal status with connection to “k” other genes in a network, was introduced to locate the core regulatory genes in the network. Accordingly, the genes with largest k-core scores and highest degrees of connection were identified as “key regulatory genes” in a network [54, 55].

### Verification by quantitative real-time PCR

To assess the reliability of our sequencing and analysis by real-time quantitative PCR (RT-qPCR), we used the same RNA samples for transcriptome sequencing. For first-strand cDNA synthesis, 1 μg of total RNA was reverse-transcribed using the Transcriptor First Strand cDNA Synthesis Kit (Roche) according to the manufacturer’s protocol. In the subsequent experiments, the cDNA samples were diluted 10 times prior to qPCR. Real-time qPCR was performed using FastStart Essential DNA Green Master (Roche) on the CFX96 Real-Time System (BioRAD, USA). The reaction was performed using the following conditions: denaturation at 95 °C for 10 min, followed by 40 cycles of amplification (95 °C for 10 s, 60 °C for 15 s and 72 °C for 20 s). Relative expression was calculated by the 2-ΔΔCt method using β-actin as the reference control [56]. All primers are listed in Additional file 10: Table S5.

## Additional files

Additional file 1: **Figure S1.** Gene structure analyses of the mapped reads on different regions of the reference duck genome. (PNG 402 kb)

Additional file 2: **Figure S2.** Analysis of the gene expression correlation between the two biological replicates of six groups (EF, EP, MF, MP, LF and LP). (PNG 280 kb)

Additional file 3: **Figure S3.** Regression analysis of gene expression fold changes (FC) obtained from quantitative PCR and RNA-Seq. (PNG 35 kb)

Additional file 4: **Figure S4.** Venn diagram of the DEGs of P, F libraries. A: Venn diagram of the DEGs of P libraries (including EP/MP, MP/LP and EP/LP); B Venn diagram of the DEGs of F libraries (including EF/MF, MF/LF and EF/LF). (PNG 329 kb)

Additional file 5: **Table S1.** The detailed information regarding the identity of the expression profiles of DEGs from MPvsEP, LPvsMP and LPvsEP. (XLS 1357 kb)

Additional file 6: **Table S2.** KEGG pathway functional enrichment analysis related with plumulaceous feather development of the DEGs of MP/EP, LP/MP and LP/EP. (DOCX 16 kb)

Additional file 7: **Figure S5.** Pathway-act-network analysis of the plumulaceous development in Cherry Valley duck. (PNG 228 kb)

Additional file 8: **Table S3.** The detailed information regarding the identity of the expression profiles of DEGs from MFvsEF, LFvsMF and LFvsEF. (XLS 1656 kb)

Additional file 9: **Table S4.** KEGG pathway functional enrichment analysis related with flight feather development of the DEGs of MF/EF, LF/MF and LF/EF. (DOCX 16 kb)

Additional file 10: **Table S5.** Primers used for qPCR validation. (DOCX 12 kb)

## Abbreviations

BMP: Bone morphogenesis protein
CAMs: Cell adhesion molecules
DEGs: Differentially expressed genes
EF: Early growth flight feathers
EP: Early growth plumulaceous feathers
FC: Fold change
FGF: Fibroblast growth factor
FPKM: Fragments per kilobase of transcript per million mapped reads
FZD: Frizzled protein
GO: Gene ontology
HOX: Homeobox
Jak-STAT: Janus kinase and the signal transducer and activator of transcription
KEGG: Kyto encyclopaedia of genes and genomes
LF: Late growth flight feathers
LP: Late growth plumulaceous feathers
MAPK: Mitogen-activated protein kinase
MF: Middle growth flight feathers
MP: Middle growth plumulaceous feathers
PDGFC: Platelet-derived growth factor C
RT-qPCR: Real-time quantitative PCR
TGF-beta: Transforming growth factor-beta; NF-kappaB
VEGF: Vascular endothelial growth factor
