# Supplementary figures and images for: Dynamic transcriptome profiling towards understanding the morphogenesis and development of diverse feather in domestic duck
Source: BMC Genomics. 2018 May 24;19:391. doi: 10.1186/s12864-018-4778-7 (PMC5968480; doi:10.1186/s12864-018-4778-7)

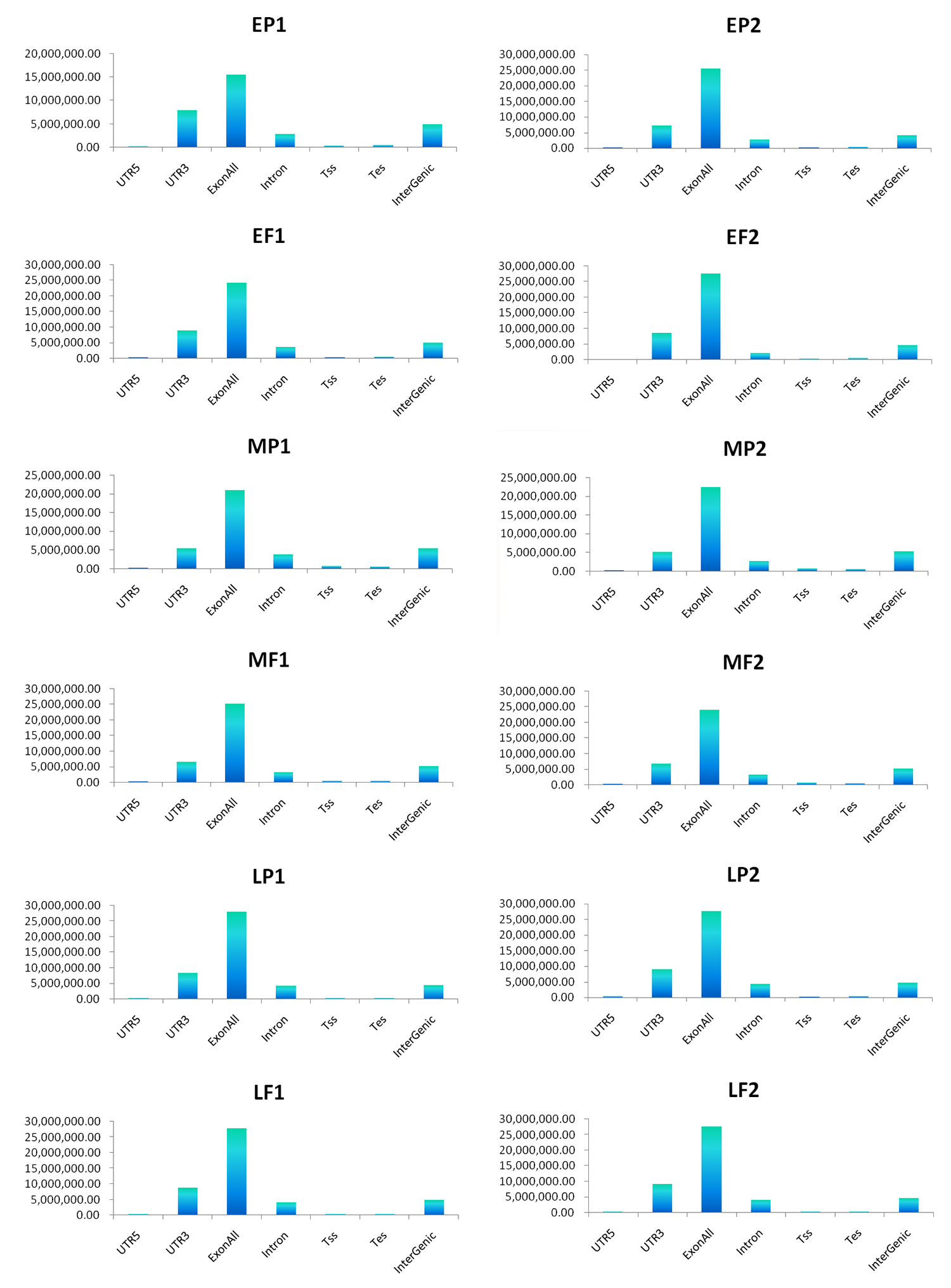

Supplement: Supplementary file 1 — Figure S1. Gene structure analyses of the mapped reads on different regions of the reference duck genome. (PNG 402 kb) [file 12864_2018_4778_MOESM1_ESM.png]

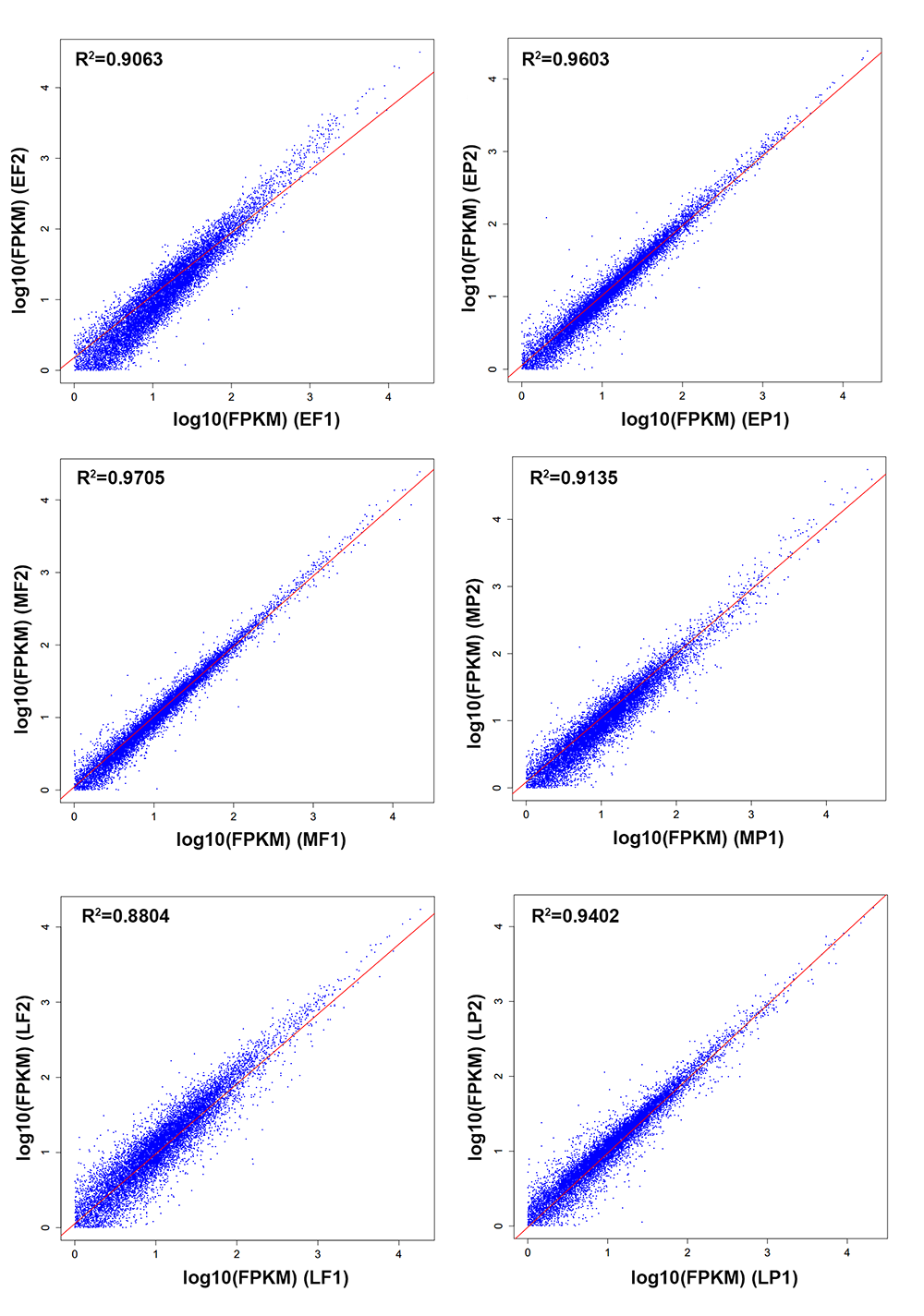

Supplement: Supplementary file 2 — Figure S2. Analysis of the gene expression correlation between the two biological replicates of six groups (EF, EP, MF, MP, LF and LP). (PNG 280 kb) [file 12864_2018_4778_MOESM2_ESM.png]

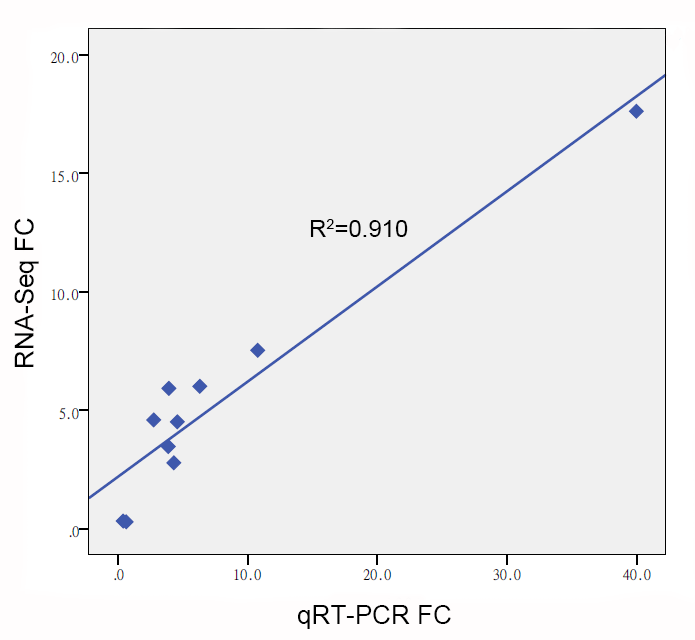

Supplement: Supplementary file 3 — Figure S3. Regression analysis of gene expression fold changes (FC) obtained from quantitative PCR and RNA-Seq. (PNG 35 kb) [file 12864_2018_4778_MOESM3_ESM.png]

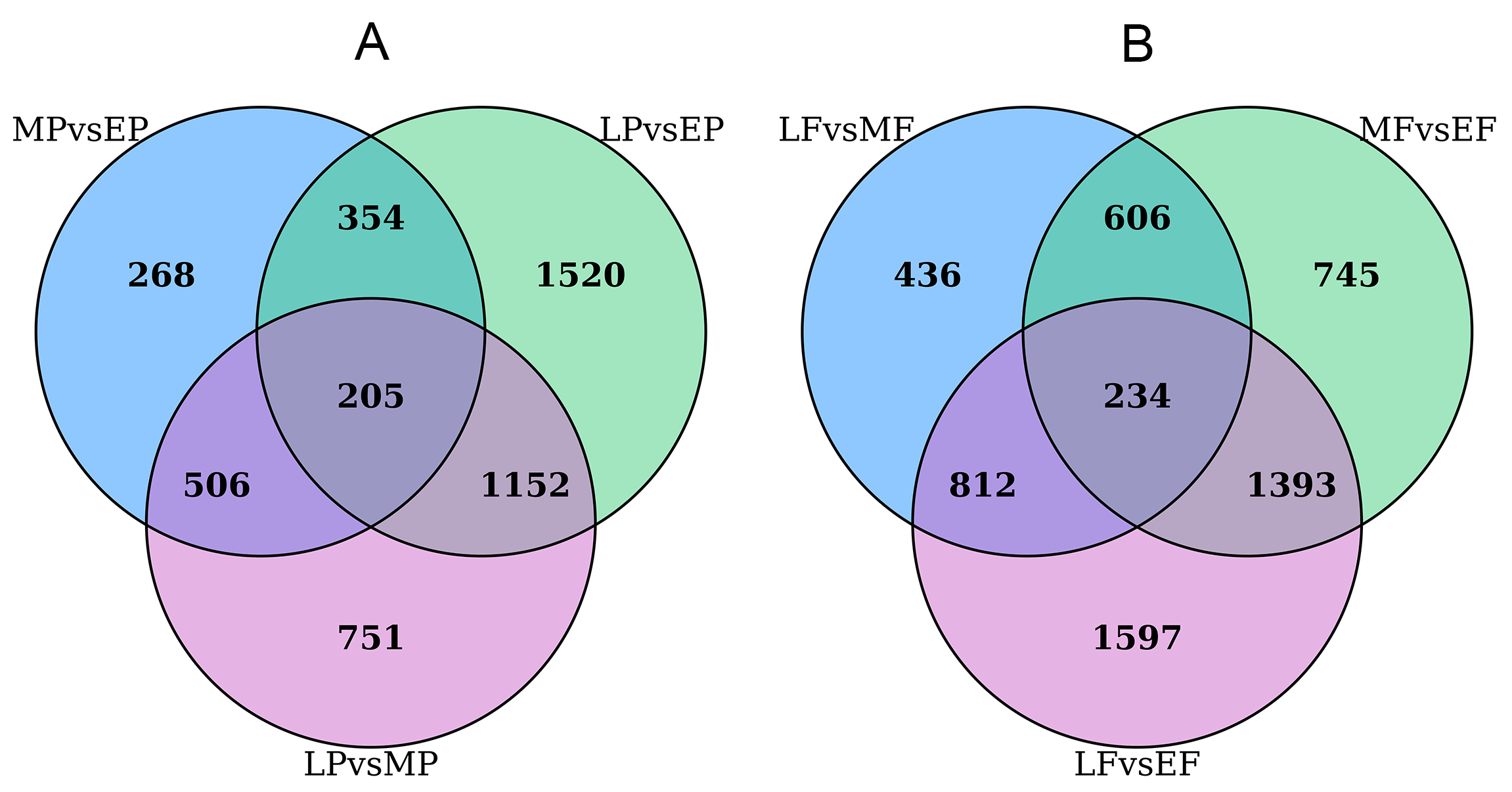

Supplement: Supplementary file 4 — Figure S4. Venn diagram of the DEGs of P, F libraries. A: Venn diagram of the DEGs of P libraries (including EP/MP, MP/LP and EP/LP); B Venn diagram of the DEGs of F libraries (including EF/MF, MF/LF and EF/LF). (PNG 329 kb) [file 12864_2018_4778_MOESM4_ESM.png]

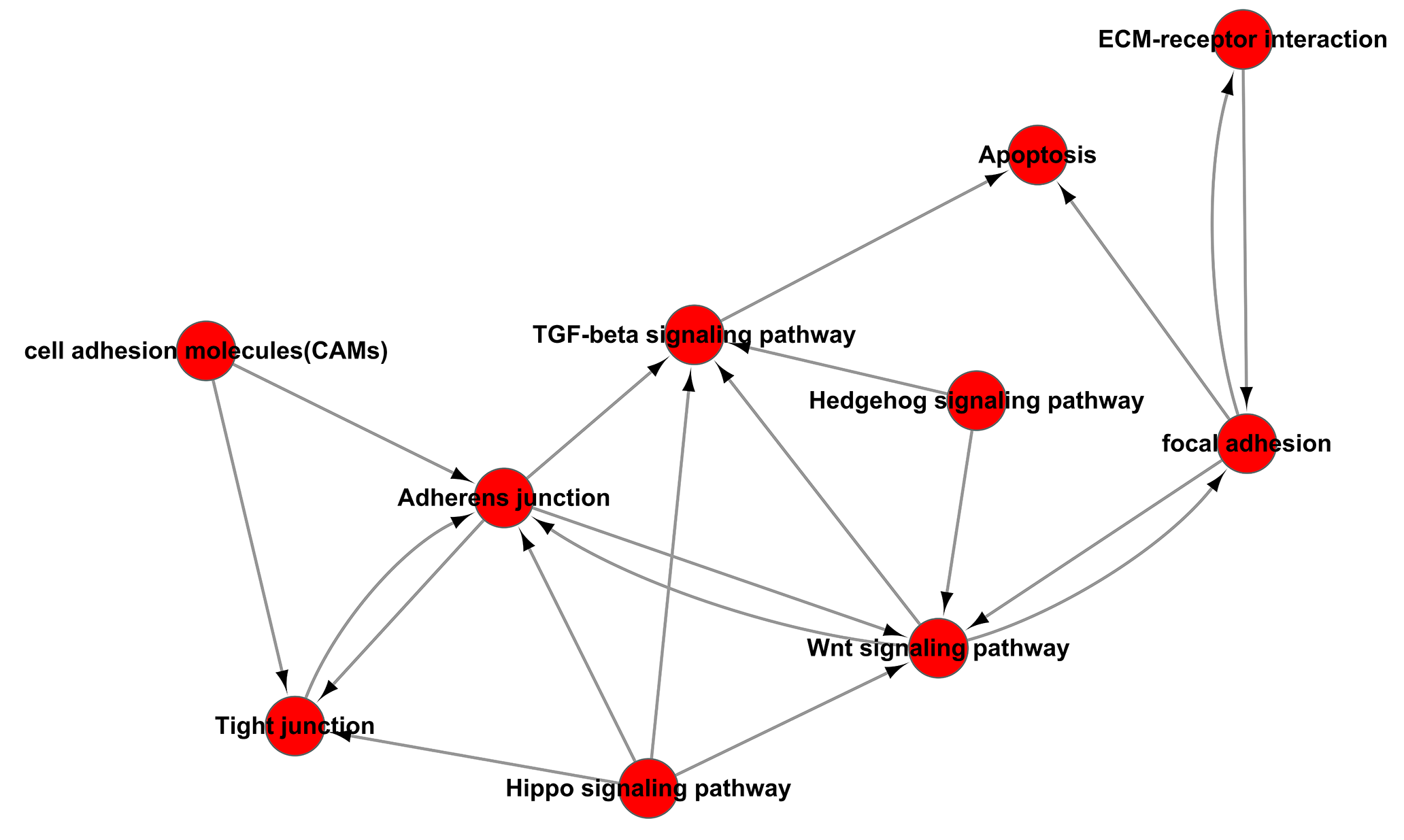

Supplement: Supplementary file 7 — Figure S5. Pathway-act-network analysis of the plumulaceous development in Cherry Valley duck. (PNG 228 kb) [file 12864_2018_4778_MOESM7_ESM.png]
