# Supplementary material for: Dynamic transcriptome profiling towards understanding the morphogenesis and development of diverse feather in domestic duck
Source: BMC Genomics. 2018 May 24;19:391. doi: 10.1186/s12864-018-4778-7 (PMC5968480; doi:10.1186/s12864-018-4778-7)
Supplement: Supplementary file 6 — Table S2. KEGG pathway functional enrichment analysis related with plumulaceous feather development of the DEGs of MP/EP, LP/MP and LP/EP. (DOCX 16 kb) [file 12864_2018_4778_MOESM6_ESM.docx]

Table S3 KEGG pathway functional enrichment analysis related with flight feather development of the DEGs of MFvsEF, LFvsMF and LFvsEF

| Tissue comparison | Pathway Term | Enricnment score | P-value |
| --- | --- | --- | --- |
| MFvsEF | MAPK signaling pathway | 1.37 | 0.0300 |
|  | VEGF signaling pathway | 1.60 | 0.0485 |
| LFvsMF | Jak-STAT signaling pathway | 2.41 | 5.59E-05 |
|  | Focal adhesion | 1.77 | 0.0014 |
|  | VEGF signaling pathway | 2.14 | 0.0048 |
|  | MAPK signaling pathway | 1.60 | 0.0053 |
|  | ECM-receptor interaction | 2.00 | 0.0072 |
|  | TNF signaling pathway | 1.99 | 0.0087 |
|  | Cell adhesion molecules (CAMs) | 1.66 | 0.0275 |
|  | NF-kappa B signaling pathway | 1.80 | 0.0311 |
|  | Adherens junction | 1.71 | 0.0339 |
| LFvsEF | ECM-receptor interaction | 1.96 | 0.0007 |
|  | Jak-STAT signaling pathway | 1.63 | 0.0064 |
|  | Focal adhesion | 1.42 | 0.0117 |
|  | NF-kappa B signaling pathway | 1.64 | 0.0201 |
|  | TNF signaling pathway | 1.59 | 0.0255 |
