# Supplementary material for: Dynamic transcriptome profiling towards understanding the morphogenesis and development of diverse feather in domestic duck
Source: BMC Genomics. 2018 May 24;19:391. doi: 10.1186/s12864-018-4778-7 (PMC5968480; doi:10.1186/s12864-018-4778-7)
Supplement: Supplementary file 9 — Table S4. KEGG pathway functional enrichment analysis related with flight feather development of the DEGs of MF/EF, LF/MF and LF/EF. (DOCX 16 kb) [file 12864_2018_4778_MOESM9_ESM.docx]

Table S2 KEGG pathway functional enrichment analysis related with plumulaceous feather development of the DEGs of MPvsEP, LPvsMP and LPvsEP

| Tissue comparison | Pathway Term | Enricnment score | P-value |
| --- | --- | --- | --- |
| MPvsEP | VEGF signaling pathway | 2.03 | 0.0318 |
|  | MAPK signaling pathway | 1.55 | 0.0321 |
| LPvsMP | Focal adhesion | 1.69 | 0.0009 |
|  | MAPK signaling pathway | 1.59 | 0.0018 |
|  | Hedgehog signaling pathway | 2.26 | 0.0057 |
|  | Hippo signaling pathway | 1.66 | 0.0062 |
|  | Cell adhesion molecules (CAMs) | 1.65 | 0.0139 |
|  | ECM-receptor interaction | 1.68 | 0.0244 |
|  | TNF signaling pathway | 1.69 | 0.0258 |
|  | Gap junction | 1.64 | 0.0300 |
|  | Adherens junction | 1.60 | 0.0365 |
|  | Axon guidance | 1.50 | 0.0417 |
|  | Jak-STAT signaling pathway | 1.49 | 0.0481 |
| LPvsEP | Focal adhesion | 1.70 | 0.0004 |
|  | ECM-receptor interaction | 2.05 | 0.0008 |
|  | TNF signaling pathway | 1.96 | 0.0023 |
|  | Hippo signaling pathway | 1.55 | 0.0112 |
|  | Wnt signaling pathway | 1.44 | 0.0358 |
