# Supplementary material for: Dynamic transcriptome profiling towards understanding the morphogenesis and development of diverse feather in domestic duck
Source: BMC Genomics. 2018 May 24;19:391. doi: 10.1186/s12864-018-4778-7 (PMC5968480; doi:10.1186/s12864-018-4778-7)
Supplement: Supplementary file 10 — Table S5. Primers used for qPCR validation. (DOCX 12 kb) [file 12864_2018_4778_MOESM10_ESM.docx]

| Table S1 Primers used for qPCR validation |
| --- |

| Gene | Primer sequences (5'-3') | Product size (bp) |
| --- | --- | --- |
| *HOXB2* | F:GAGTAGACACACACGCACGA R:AGAAAGGCAAGGTGGAGAAT | 137 |
| *HOXB3* | F:AGCCTCGTATTTGCTGGACC R:TATGGCAGAACATTGGCACC | 103 |
| *HOXB4* | F:GGACTCCAACTTTGTTCACTTG R:ATCGCTGTGGGAATACTCTTC | 112 |
| *HOXB6* | F:AGAGGAGATTCTGGACCGAG R:GAGAGACAGCGTTTGCTTATC | 171 |
| *HOXD10* | F:CAGAAGTCCAGGAGAAGGAG R:CCAGTGTTTGGTGCTTAGTG | 120 |
| *MGP* | F:AATGCGCACTCTCATCATCCT R:CAGTTTGCATGAAGCCGTTG | 144 |
| *EGFL6* | F:CAAATGGAAGGACATGCGTTGA R:ACACAGTCATAATGGCCACTGA | 155 |
| *FGF13* | F:CTGAAGCAAGTGCACCTCAA R:AATCGCCACCACTCGTAAAC | 195 |
| *ACTG2* | F:CCTTCATTGGCATGGAGTCT R:CGCGATGATTTTGATCTTCA | 200 |
| *FHL2* | F:CAAACCCGATCAGTGGTCTC R:CCTCTCTTCCCTGCCTCTTT | 197 |
